# A Newly Isolated *Bacillus subtilis* Strain Named WS-1 Inhibited Diarrhea and Death Caused by Pathogenic *Escherichia coli* in Newborn Piglets

**DOI:** 10.3389/fmicb.2019.01248

**Published:** 2019-06-12

**Authors:** Yunping Du, Zhichao Xu, Guolian Yu, Wei Liu, Qingfeng Zhou, Dehong Yang, Jie Li, Li Chen, Yun Zhang, Chunyi Xue, Yongchang Cao

**Affiliations:** ^1^Biochemistry and Molecular Biology Laboratory, State Key Laboratory of Biocontrol, School of Life Science, Sun Yat-sen University, Guangzhou, China; ^2^Animal Disease Laboratory, Wen’s Group Academy, Wen’s Foodstuffs Group Co., Ltd., Xingning, China; ^3^Department of Biological Engineering, School of Biology and Food Engineering, Changshu Institute of Technology, Suzhou, China

**Keywords:** *Bacillus subtilis*, genomic analysis, biocontrol agent, newborn piglets, Escherichia coli

## Abstract

*Bacillus subtilis* is recognized as a safe and reliable human and animal probiotic and is associated with bioactivities such as production of vitamin and immune stimulation. Additionally, it has great potential to be used as an alternative to antimicrobial drugs, which is significant in the context of antibiotic abuse in food animal production. In this study, we isolated one strain of *B. subtilis*, named WS-1, from apparently healthy pigs growing with sick cohorts on one *Escherichia coli* endemic commercial pig farm in Guangdong, China. WS-1 can strongly inhibit the growth of pathogenic *E. coli in vitro*. The *B. subtilis* strain WS-1 showed typical *Bacillus* characteristics by endospore staining, biochemical test, enzyme activity analysis, and 16S rRNA sequence analysis. Genomic analysis showed that the *B. subtilis* strain WS-1 shares 100% genomic synteny with *B. subtilis* with a size of 4,088,167 bp. Importantly, inoculation of newborn piglets with 1.5 × 10^10^ CFU of *B. subtilis* strain WS-1 by oral feeding was able to clearly inhibit diarrhea (*p* < 0.05) and death (*p* < 0.05) caused by pathogenic *E. coli* in piglets. Furthermore, histopathological results showed that the WS-1 strain could protect small intestine from lesions caused by *E. coli* infection. Collectively, these findings suggest that the probiotic *B. subtilis* strain WS-1 acts as a potential biocontrol agent protecting pigs from pathogenic *E. coli* infection.

**Importance:** In this work, one *B. subtilis* strain (WS-1) was successfully isolated from apparently healthy pigs growing with sick cohorts on one *E. coli* endemic commercial pig farm in Guangdong, China. The *B. subtilis* strain WS-1 was identified to inhibit the growth of pathogenic *E. coli* both *in vitro* and *in vivo*, indicating its potential application in protecting newborn piglets from diarrhea caused by *E. coli* infections. The isolation and characterization will help better understand this bacterium, and the strain WS-1 can be further explored as an alternative to antimicrobial drugs to protect human and animal health.

## Introduction

*Bacillus* was first reported by Christian Gottfried Ehrenberg in 1835 (Sella et al., 2015). Since then, *Bacillus* has been found in a wide variety of organisms, such as pigs, and environments, such as ponds and soil (Borsodi et al., 2011; Gu et al., 2015; Wei et al., 2016). *Bacillus* is a common bacterium and is Gram-positive, rod-shaped with a size range of 0.3~22 μm × 1.2~7.0 μm, spore-forming, and aerobic-to-facultative (Cowan, 1974). A circular colony with rough, opaque, fuzzy white or slightly yellow, and jagged edges was observed by culturing *Bacillus* on nutrient agar (Lu et al., 2018). The genus *Bacillus* was initially proposed by Cohn in 1872 (Harwood, 1989), since then the genus has been expanded with many novel strains. In general, *Bacillus* can be classified into three categories based on the morphology of the spore: (1) seven species including *Bacillus subtilis* in the first genus with ovular- or pillar-shaped spores and with sporocyte that is not significantly expanded; (2) nine species including *Bacillus circulans* in the second genus with oval spore and enlarged sporocyte; and (3) *Bacillus sphaericus* in the third genus with a circular spore and enlarged sporocyte (Ruth et al., 1973).

*Bacillus* is widely used in industry, agriculture, and the medical field since a variety of functional *Bacillus* strains are extensively used in the production of industrial enzymes, bioinsecticides, antibiotics, and other products (Schallmey et al., 2004). *B. subtilis* is one of the most commonly functional strains. Currently, several studies have confirmed that *B. subtilis* has great application value in animal husbandry. It was reported that pig feed with *B. subtilis* natto could significantly improve meat quality and flavor (Sheng et al., 2016). Liu et al. (2017) also demonstrated that dietary corn bran fermented by *B. subtilis* MA139 could decrease gut cellulolytic bacteria and microbiota diversity in finishing pigs. In addition, *B. subtilis* as delivery vectors is also used to develop vaccines against TGEV in pigs (Mou et al., 2016). Additionally, more functions such as the antimicrobial activity of *B. subtilis* have been identified with the discovery and isolation of new strains (Sato et al., 2001; Phelan et al., 2013; Piewngam et al., 2018; Bartolini et al., 2019; Swartzendruber et al., 2019). For example, it has been found by Bartolini et al. (2019) that the stress-responsive alternative sigma factor (SigB) of *B. subtilis* enhanced antifungal proficiency by increasing the synthesis of lipopeptide surfactin. In addition, Piewngam proved that fengycin-producing *Bacillus* could inhibit *Staphylococcus aureus* colonization in mice (Piewngam et al., 2018).

The accurate identification of a new bacterium is an essential part of studying its function. The traditional techniques to identify a new bacterium are mainly based on the use of selective media by observing bacterial colony characteristics and morphology and by biochemical tests (Lu et al., 2018). Although these methods can preliminarily identify the type of the new strain, the detailed information such as genus, genomic composition, and functional proteins remains unclear. After the conserved bacterial genomic regions are amplified and sequenced, the genus of the bacteria will be accurately identified by homology analysis with the sequences from GenBank (Wellinghausen et al., 2009). These methods contribute to rapid and accurate identification of new bacterial strains.

Despite various applications of *B. subtilis* in many fields, detailed information underlying resistance against intestinal infections in the pig remains unclear. In this study, we isolated one strain of *B. subtilis* WS-1 from apparently healthy pigs raised with sick cohorts on one *Escherichia coli* endemic commercial pig farm in Guangdong, China and analyzed its genome. In addition, *B. subtilis* WS-1 was identified to inhibit the growth of pathogenic *E. coli in vitro* and *in vivo*, which implied that the isolate of *B. subtilis* WS-1 could have potential usage in the future.

## Materials and Methods

### Bacterial Strain and Experimental Newborn Piglets

*E. coli* strain 4–1, belonging to serogroup O149:K88 and containing LT and ST genes, was isolated from the same commercial pig farms as *B. subtilis* WS-1 and was confirmed to be highly pathogenic to the newborn piglets. Four-day-old crossbred (Duroc × Landrace × Big White) healthy conventional female newborn piglets without diarrheic symptoms were procured from Wen’s Foodstuffs Group Co, Ltd. (Guangdong, China). All piglets were fed a mixture of skim milk powder (Inner Mongolia Yili Industrial Group Co., Ltd., China) with warm water. The animal study was approved by the Institutional Animal Care and Use Committee of the Sun Yat-sen University (Guangdong, China), and animals were treated in accordance with the regulations and guidelines of this committee.

### Reagents and Culture Medium

The Tryptic Soy Broth (TSB) medium and Tryptic Soy Agar (TSA) medium were purchased from Becton, Dickinson and Company (USA). The Oxford cup and Gram Stain Kit were purchased from Guangzhou Heyue Biotechnology Co., Ltd. (China). Endospore Stain Kit was purchased from Solarbio Company (China). TIANamp Bacteria DNA Kit was purchased from TIANGEN Biotech Co., Ltd. (Beijing, China). The Premix Taq™ (LA Taq™ Version 2.0 plus dye) and pMD19-T were purchased from Takara (Dalian, China).

### Bacteria Isolation and Inoculum Preparation

Fresh pig feces (10 g) were collected from apparently healthy pigs growing with diarrheic cohorts on one *E. coli* endemic commercial pig farm in Guangdong, China. Prior to isolation, pig feces were mixed with 90 μl sterile 1 × phosphate buffer saline (PBS; pH 7.4), then incubated in an orbital shaker incubator (Shanghai Bluepard Instruments Co., Ltd., China) at room temperature with a shaking speed of 180 rpm for 30 min, then serial diluted up to 10^−7^ with a sterile distilled water. Isolation of bacteria from this mixture was done with a serial dilution technique in TSA medium (BD, USA). Bacteria were purified by repeated streaking and single colony culture at 37°C for 17–24 h. A total of 35 unknown bacterial isolates were recovered in TSB medium (BD, USA). Exponential phase growing cultures were washed twice with sterile 1 × PBS and maintained at 4°C until use.

### Screening of the Antimicrobial Isolates Using the Oxford Cup Method

The Oxford cup method was performed as previously described with some modifications (Bian et al., 2016). Briefly, the culture fluid of *E. coli* strain 4–1 was mixed with the TSA medium at 50°C and poured into a bacterial culture dish. The Oxford cups containing 80 μl (7.2 × 10^8^ CFU/ml) culture fluid of 35 unknown bacteria strains were affixed to the uniform coating on the medium, placed at 37°C for 18–24 h, and the inhibitory rings were observed and measured with a Vernier caliper (Guangzhou Heyue Biotechnology Co., Ltd., China). The Oxford cups containing culture fluid without bacteria were used as control.

### Identification of the No. 1 Unknown Bacterial Strain

(1) The No. 1 unknown bacterial strain was examined for being a member of the family *Bacillus* by means of endospore staining according to the manufacturer’s instruction (Solarbio Company, China), Gram staining (Lu et al., 2018), and biochemical trait test (Cowan, 1974) and further by enzyme activity (Cowan, 1974). (2) Molecular identification of the *Bacillus* isolate by PCR and total DNA of the No. 1 unknown bacterial strain were prepared according to the manufacturer’s instruction (TIANGEN Company, China). The universal bacterial primers for the 16S rRNA gene of *Bacillus* (sense: 5′-AGAGTTGATCCTGGCTAAG-3′; antisense: 5′-GGTTACCTTGTTACGACTT-3′) were designed with reference to a previous publication (Lu et al., 2018) and were synthesized by Sangon Company (Shanghai, China). The PCR was performed in a volume of 50 μl containing 1 μl of DNA, 25 μl Premix Taq™ (LA Taq™ Version 2.0 plus dye), upstream and downstream primer (50 μmol/L) each at 1 μl and 22 μl ddH_2_O. The thermal cycling parameters were as follows: 94°C for 5 min; 35 cycles of 94°C for 1 min, 55°C for 1 min, and 72°C for 1 min; and a final extension at 72°C for 15 min. The positive PCR products were cloned into the pMD19-T (TaKaRa, Dalian) and sequenced by Sangon Company (Shanghai, China). Sequence alignments of 16S rRNA of different *Bacillus* species were performed using the DNAStar Lasergene 7.0. A genome homology analysis and phylogenetic trees were constructed by using the maximum likelihood method with MEGA 5 software^1^ based on the 16S rRNA nucleotide sequences of 14 *Bacillus* strains from different countries.

### Genome Sequencing and Bioinformatics Analysis

After we successfully identified the No. 1 unknown strain as a member of *B. subtilis*, which was thereafter named as *B. subtilis* WS-1, the complete genome was sequenced by the PacBio platform (Single Molecule, Real-Time technology) (Magigene, Guangdong) as previously described with some modifications (Cameron et al., 2015; Tanizawa et al., 2015; Carrión et al., 2018). Briefly, the bacterial genomic DNA was extracted as described above. DNA integrity and purity were monitored on 1% agarose gels, and the concentration and purity of DNA were measured using Qubit 2.0 (Thermo Fisher Scientific Waltham, USA) and Nanodrop one (Thermo Fisher Scientific Waltham, USA) at the same time. Then, the qualified genomic DNA was fragmented with G-tubes (Covaris) and end-repaired to prepare SMRTbell DNA template libraries (with a fragment size of >10 kb selected by bluepippin system) according to the manufacturer’s specification (PacBio, Menlo Park, USA). Library quality was detected by Qubit 3.0 Fluorometer (Life Technologies, Grand Island, NY, USA), and average fragment size was estimated on an Agilent 4,200 (Agilent, Santa Clara, CA, USA). SMRT sequencing was performed on the Pacific Biosciences RSII sequencer (PacBio, Menlo Park, USA) according to standard protocols. The low-quality reads were filtered by the SMRT 2.3.0 and assembled to generate one contig without gaps after sequencing. For the genome component prediction, the whole genome sequence was performed by Gene Marks for coding gene prediction (Besemer et al., 2001), diamond and BLAST for gene annotation, and PHAST for pre-phage prediction (Zhou et al., 2011). Related functional proteins were analyzed by BLAST. The genome overview was created by Circos (Krzywinski et al., 2009) to show the annotation information. Genomic synteny was analyzed by MUMmer software (Delcher et al., 2002) based on the alignment results with *B. subtilis* (GenBank no: AL009126.3).

### Measurement of Bacterial Growth

To determine the growth rate of *B. subtilis* WS-1 (3.4 × 10^4^), the strain was grown in TSB medium at 37°C for 48 h with agitation, and the CFU was determined at 0, 6, 12, 18, 24, 30, 36, 42, and 48 h.

### Experimental Infection With the *E. coli* 4–1 Strain After *B. subtilis* Strain WS-1 Treatment in Conventional Newborn Piglets

Twelve newborn piglets were randomly divided into two groups (6 piglets/group) and were housed in two separate rooms. Prior to inoculation, newborn piglets were confirmed negative for the major porcine enteric viruses (PDCoV, PEDV, TGEV, and PRoV) by testing the rectal swabs collected from the newborn piglets on day 1 as previously described (Xu et al., 2018). On day 0, newborn piglets in group 1 were orally inoculated with 5 ml/day of TSB medium for 3 days. Newborn piglets in group 2 were orally inoculated with 5 ml/day of TSB medium containing a total of 5 × 10^9^ CFU of the *B. subtilis* strain WS-1 (1 ml of medium contained 1 × 10^9^ CFU of *B. subtilis* strain WS-1) for 3 days. Afterward, all piglets were orally challenged with 5 ml TSB medium containing 1 × 10^10^ CFU of the *E. coli* 4–1 strain. The piglets were observed daily for clinical signs of diarrhea and lethargy. One piglet from each group was necropsied at 3 days post challenge (d.p.c). At necropsy, the fresh duodenum, jejunum, and ileum were collected and fixed by 10% formalin for histopathology analysis. In addition, the mortality rate of newborn piglets in different treatment groups was recorded daily from day 1 to day 6 after challenge for protection rate analysis.

### Histological Staining

Histological staining was performed as previously described (Xu et al., 2018). Briefly, tissue samples of the duodenum, jejunum, and ileum of the piglets from the *B. subtilis* strain WS-1 treatment and control groups were routinely fixed in 10% formalin for 36 h at room temperature and then dehydrated in graded ethanol, embedded in paraffin, cut in 5-μm sections, and mounted onto glass slides. After the sections were deparaffinized, rehydrated, and stained with hematoxylin and eosin (H&E), the slides were examined and analyzed with conventional light microscopy (Nikon, Japan).

### Statistical Analysis

Statistical comparisons were performed using GraphPad Prism software. The significance of the differences between the treatment group and control in the inhibitory rings, diarrhea rate, and survival rate was determined by ANOVA and Mann-Whitney accordingly.

## Results

### Ten Unknown Bacterial Strains Isolated From the Feces of Apparently Healthy Pigs Inhibit the Growth of Pathogenic *E. coli in vitro*

**Figure 1:**
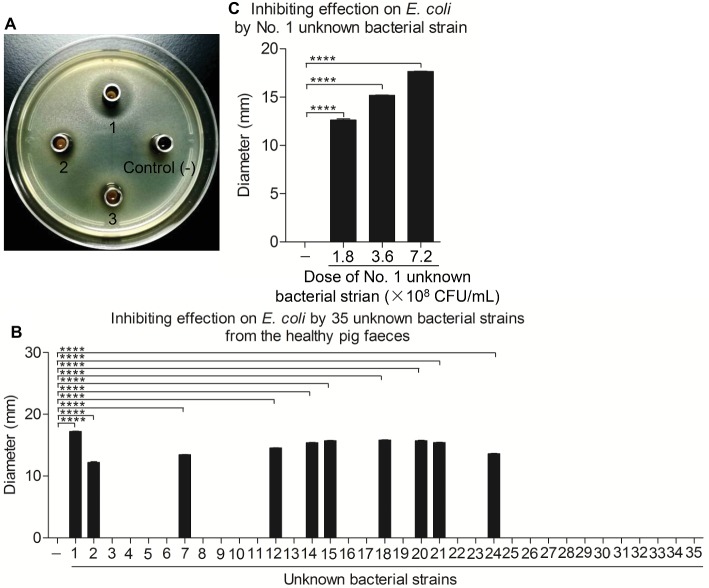
Ten unknown bacterial strains from healthy pig feces significantly inhibited the growth of *E. coli in vitro*. **(A,B)** Inhibiting effect on *E. coli* of total 35 unknown bacterial strains isolated from the healthy pig feces. *E. coli* strain 4–1 was maintained on bouillon agar medium (10^8^ CFU/ml); the Oxford cup with 80 μl single bacterial culture fluid was placed in the middle. After 24 h, the inhibitory ring was observed and measured with ruler. **(C)** The No. 1 unknown bacterial strain significantly inhibited the growth of *E. coli*. *E. coli* strain 4–1 was maintained on bouillon agar medium (10^8^ CFU/ml); the Oxford cups with 80 μl of No. 1 unknown bacterial culture fluid containing different amounts of 1.8 × 10^8^, 3.6 × 10^8^, and 7.2 × 10^8^ CFU/ml were placed in the middle. The inhibitory rings were observed and measured with a ruler. Data are representative of three independent experiments. Data are represented as mean ± SD, *n* = 3. ****p < 0.0001.

Some apparently healthy piglets without any symptoms of diarrhea were found on one *E. coli* endemic commercial pig farm in Guangdong, China. To determine the protective agent against the diarrhea outbreak in this pig farm, fresh feces from one healthy pig were collected and used to isolate the bacteria. Of the 10 g of feces examined, 35 unknown bacterial strains were isolated. Furthermore, we found that 10 unknown bacterial strains could markedly inhibit the growth of *E. coli* by the Oxford cup method (Figures 1A,B). Of them, the No. 1 unknown bacterial strain had the best antibacterial effect in a dose-dependent manner (Figures 1B,C).

### A Strain of *B. subtilis* Was Identified

**Figure 2:**
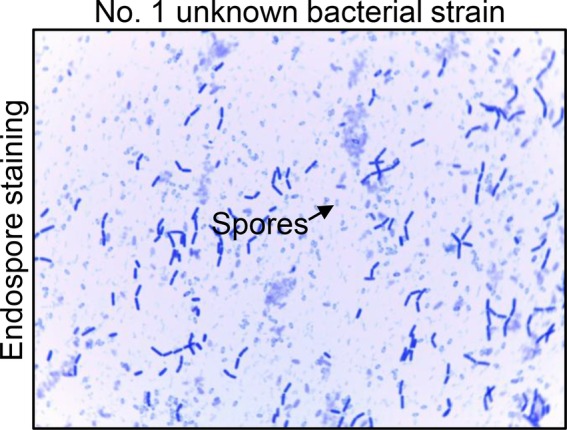
The endospore staining of No. 1 unknown bacterial strain. Endospore staining to analyze the characteristic of No. 1 unknown bacterial strain. The arrows show the forming spores of this unknown bacterium.

**Figure 3:**
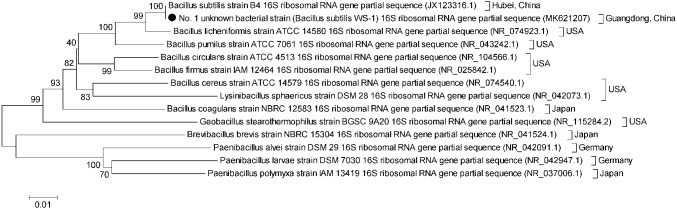
Phylogenetic tree constructed on the basis of the 16S rRNA gene from *B. subtilis* strain WS-1 and 13 *B. subtilis* strains from different countries. The dendrogram was constructed using the neighbor joining method in the MEGA software package, version 5. Reference sequences obtained from GenBank are indicated by strain name. The scale bar represents 0.01 nucleotide substitutions per site.

The No. 1 unknown bacterial strain was identified as *Bacillus* by morphological and biochemical examinations. The isolate displayed the morphology of *Bacillus* by visual and microscopic observations and was shaped as Gram-positive (data not shown) *Bacillus*, which could form spores (Figure 2). A biochemical test was further employed to analyze the characteristic of this bacterium. The results clearly showed that the strain was up to 99% in consistency with the standard of *Bacillus* (Table 1). These results indicated that the No. 1 unknown bacterial strain was a strain of *Bacillus*. To determine the species of *Bacillus* of this strain, we further analyzed the 16S rRNA by sequencing the PCR-amplified product. As shown in Figure 3,^2^ the No. 1 unknown bacterial strain shared 99% identity with *B. subtilis* based on the sequence of 16S rRNA. Phylogenetic analysis showed that the No. 1 unknown bacterial strain was clustered into a clade with *B. subtilis* B4 from Hubei, China. Taken together, the No. 1 unknown bacterial strain belongs to *B. subtilis* and was named WS-1.

**Table 1:** Biochemical traits of the *Bacillus* isolate (*B. subtilis* strain WS-1).

Test	Result	Test	Result	Test	Result
Glycerin	+	Inositol	+	Inulin	+
Erythritol	−	Mannitol	+	Melezitose	−
D-arabinose	−	Sorbitol	+	Raffinose	+
L-arabinose	+	α-methyl-D mannoside	−	Starch	+
Ribose	+	α-methyl-D glucoside	+	Glycogen	+
D-xylose	+	N-acetyl-glucosamine	−	Xylitol	−
L-xylose	−	Amygdalin	+	Gentian disaccharide	−
Amoxicol	−	Arbutin	+	D-Turanose	+
β-methyl-D-xyloside	−	Esculin	+	D-lyxose	−
Galactose	−	2-Hydroxybenzyl alcohol	+	D-Tagatose	−
Glucose	+	Cellobiose	+	D-arabinitol	−
fructose	+	Maltose	+	L-arabinitol	−
Mannose	+	Lactose	−	Gluconate	−
Sorbose	−	Melibiose	+	2-keto-gluconate	−
L-Rhamnose	−	Sucrose	+	5-keto-gluconate	−
Dulcitol	−	Trehalose	+		

### Complete Genome Sequencing and Analysis of *B. subtilis* Strain WS-1

**Figure 4:**
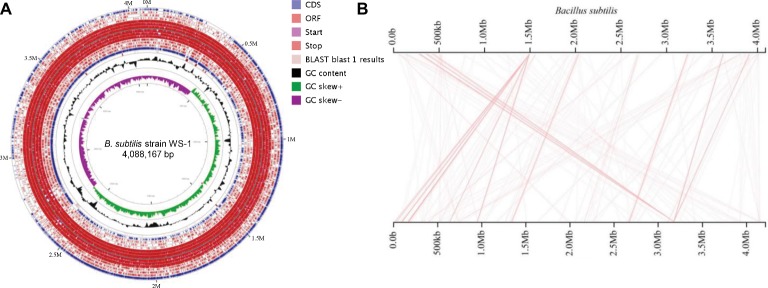
Genome visualization and genomic synteny of the *B. subtilis* strain WS-1. **(A)** Genome visualization of *B. subtilis* strain WS-1. The genome of *B. subtilis* strain WS-1 was sequenced and analyzed using the PacBio platform. **(B)** Genomic synteny of *B. subtilis* strain WS-1 was analyzed based on the alignment results with *B. subtilis*.

The complete genome of *B. subtilis* strain WS-1 was acquired and uploaded onto GenBank (No. CP024921). The genome of *B. subtilis* strain WS-1 has a size of 4,088,167 bp (Figure 4A), with G + C content being 43.8%. Some genes encoded by this strain of *Bacillus* have protease, lipase, and amylase activity (Table 2), indicating that the WS-1 strain might have antimicrobial activity. In addition, approximately 89% of nucleotides were predicted as protein-coding regions, and 86.8% (3,704) of the open reading frames were annotated with known proteins. Some putative proteins were predicted to be associated with antimicrobial or probiotic activity by the Swiss-Prot database (Table 3). We also confirmed that the WS-1 strain contained sporulation genes like *CgeA*, *CotB*, and *CotZ* (data not shown). These data confirmed that the WS-1 strain shares 100% genomic synteny with *B. subtilis* (Figure 4B).

**Table 2:** Enzyme activity analysis of *B. subtilis* strain WS-1.

Variety	Nucleotide position*	Enzyme activity (U/ml)
Protease	(−) 210183-211151, 684509-686197, 725622-727559, 758838-761258, 1122374-1122967, 2696411-2697742, 2813385-2814671, 2814668-2815948, 2829137-2830327, 2847680-2848948, 2882897-2884300, 2884317-2884862, 3384242-3385801, 3415417-3418101, 3467502-3468782, 3906905-3907594; (+) 516616-517818, 1045409-1046851, 1283746-1285122, 1364459-1365088, 1450962-1451639, 1783051-1784313, 1784465-1786123, 1786304-1788628, 1804131-1804667, 1812254-1813120, 1895220-1895846, 2095165-2095638, 2134016-2135539, 2187051-2188331, 2300552-2301154, 2306573-2307229, 2439721-2441121, 2710268-2711596, 3031354-3032919, 3135226-3137355, 3185998-3186957, 3466390-3467463, 3498168-3498779, 3899889-3900581	40.1
Lipase	(−) 163484-164155, 3661219-3661836, 4066571-4067212; (+) 2415760-2416569	28.6
Amylase	(−) 126705-128684	158.2

* *Nucleotide position is numbered based on *B. subtilis* WS-1 strain (CP024921)*.

*(+): Positive-sense strand; (−): negative-sense strand*.

**Table 3:** Functional proteins prediction of *B. subtilis* strain WS-1.

Name	Nucleotide position*	Number
Polyketides	(−) 2714457-2722088, 2722103-2738647, 2738637-2751425, 2751441-2754173, 2754134-2764267, 2764255-2765067, 2765212-2765610, 2765607-2780180, 2780225-2780974, 2781014-2781793, 2781781-2783043, 2783044-2784291, 2784579-2786513, 2786501-276881, 278678-2787852, 2788356-2789222, 2790462-2791079; (+) 3033214-3034185	18
Lipopeptides	(−) 20315-21268, 90550-91134, 132322-133203, 147031-147990, 164284-165411, 600839-601852, 962123-963040, 1243143-1243943, 1249990-1250721, 1428128-1429255, 1570007-1571287, 1865158-1865979, 2955757-2956074, 2956062-2956208, 3207178-3208827, 3350410-3352047, 3463183-3464229,3586153-3586992, 3608704-3609702, 3784560-3785786, 3824696-3825895, 4012394-4013515, 4063703-4065709 (+) 44837-45643, 87329-88180, 267938-269866, 272995-273948, 608703-609497, 650251-651399, 683398-684237, 740201-741358, 1074976-1075785, 1113395-1114648, 1160684-1161949, 1207777-1208697, 1264758-1265702, 1306601-1307425, 1318172-1318681, 1318687-1319430, 1523167-1524108, 1581821-1583335, 1660893-1661702, 1661692-1662528, 1726112-1727431, 1965356-1966111, 2058797-2059528, 2125691-2126728, 2214635-2215402, 2415760-2416569, 2437636-2438457, 2457696-2458565, 2676968-2677603, 3500643-3501521, 3651495-3652505, 3745050-3746072	55
Flagella	(−) 1138642-1139100, 2861243-2862133, 2862130-2863230, 2863230-2865263, 2865296-2866378, 2866378-2867157, 2867165-2867410, 2867449-2868114, 2868107-2868766, 2869169-2870305, 2870295-2871293, 2871327-2871749, 2871746-2871961, 2872001-2872762, 2872817-2873239, 2873236-2874699, 2875337-2875780, 2877096-2877857, 2877841-2878857, 2878870-2880480, 2880526-2880852, 2880857-2881312, 2881309-2881704, 2892718-2892999; (+) 921972-922808, 922854-923651, 1029455-1030963, 1030974-1031870, 1032513-1032944, 1034454-1034783, 1034801-1034947, 1034977-1036296, 1036318-1036719, 1625025-1625846, 1625836-1626564, 3137648-3138550, 3138522-3139307	37
Exopolysaccharide biosynthesis	(+) 556979-558043, 1143948-1144784, 1149168-1150202, 1152318-1152968, 1153006-1154139, 1154136-1155086	6
Enolase	(−) 3144704-3145948; (+) 3395921-3397036	2

* *Nucleotide position is numbered based on *B. subtilis* WS-1 strain (CP024921)*.

*(+): Positive-sense strand; (−): negative-sense strand*.

### The Growth Rate of *B. subtilis* Strain WS-1 *in vitro*

**Figure 5:**
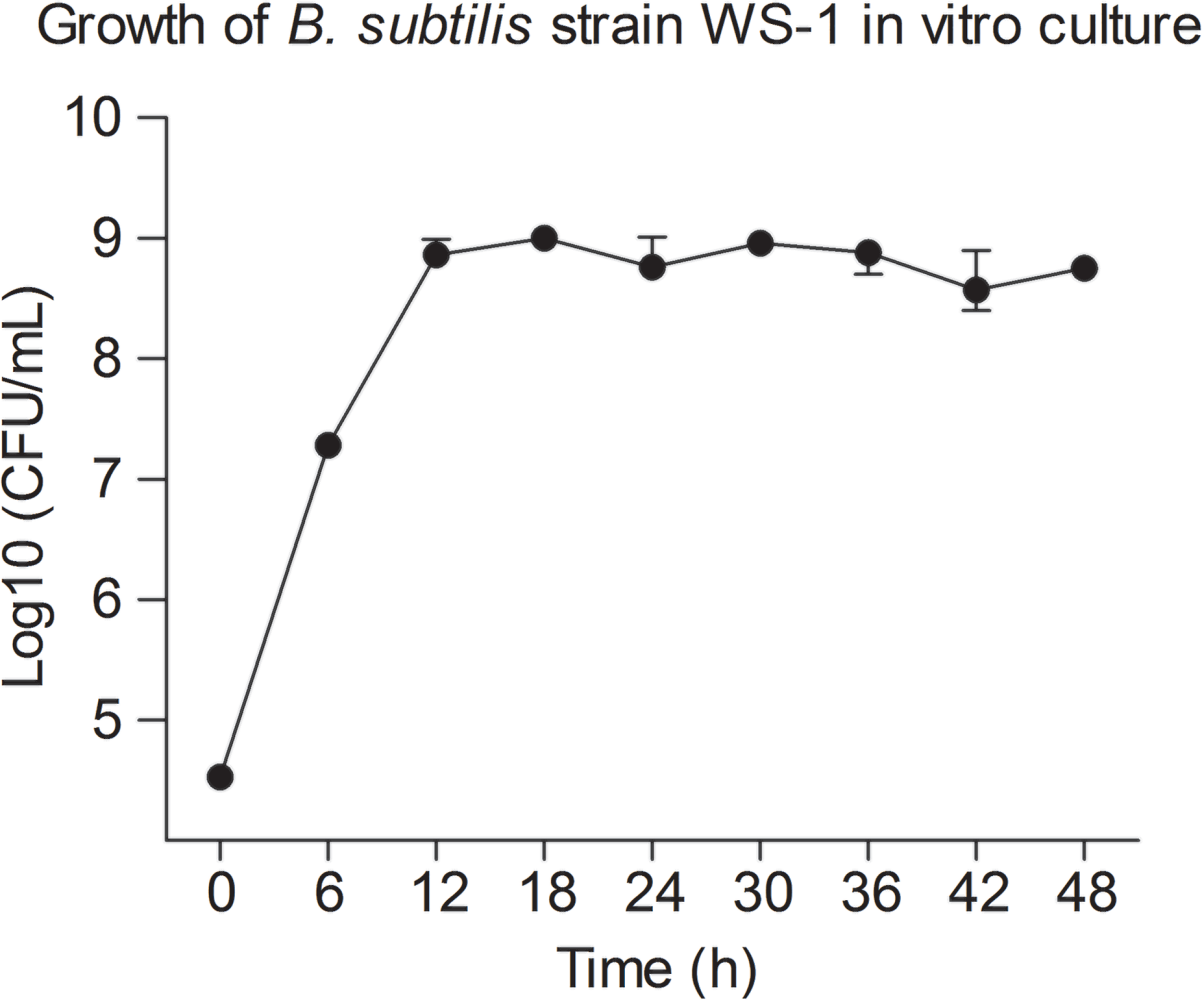
Measurement of bacterial growth. The growth curves of *B. subtilis* strain WS-1. Bacteria were grown in bouillon fluid medium at 37°C for 48 h with agitation, and the Colony Forming Unit (CFU) was determined at 0, 6, 12, 18, 24, 30, 36, 42, and 48 h. The data are representative of three independent experiments. Data are represented as mean ± SD, *n* = 3.

To determine the growth rate *in vitro*, we inoculated *B. subtilis* strain WS-1 in TSB medium and detected the living bacterial count at corresponding time points. As shown in Figure 5, the *B. subtilis* strain WS-1 grew exponentially post inoculation and reached a plateau 12 h post inoculation, which lasted for at least 36 h, indicating that *B. subtilis* strain WS-1 could adapt to the TSB medium *in vitro*.

### *B. subtilis* Strain WS-1 Inhibits Diarrhea and Death Caused by *E. coli* in Newborn Piglets

**Figure 6:**
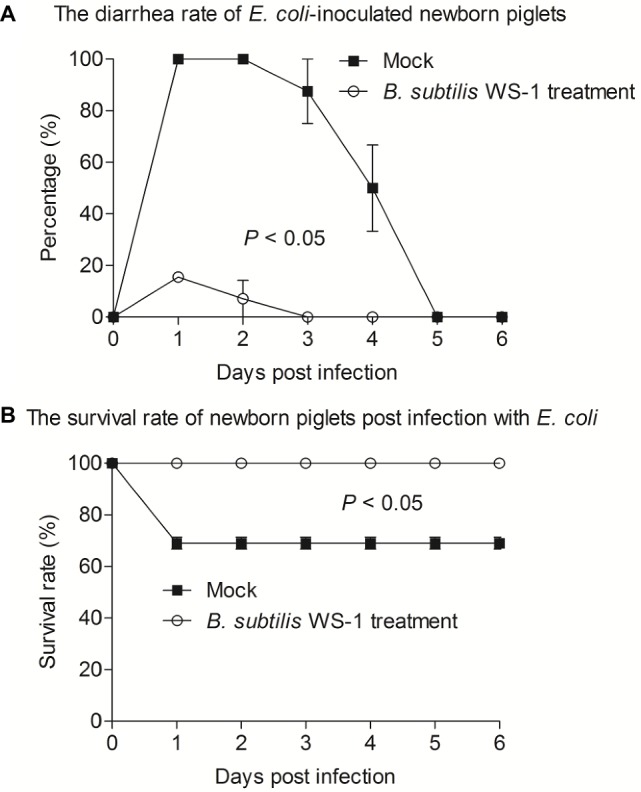
*B. subtilis* strain WS-1 inhibited diarrhea and death caused by *E. coli* in newborn piglets. Newborn piglets were first orally fed with TSB medium or TSB medium containing *B. subtilis* strain WS-1. At 3 days, all piglets were orally challenged with *E. coli* strain 4–1. The diarrhea rate **(A)** and survival rate **(B)** of newborn piglets post-challenge with *E. coli* between the control group and the *B. subtilis* strain WS-1 treatment group were recorded daily from the first day to the sixth day after challenge. The data are representative of two independent experiments. Data are represented as mean ± SD, *n* = 6 or *n* = 7.

Since *B. subtilis* strain WS-1 was confirmed to significantly inhibit the growth of *E. coli in vitro*, we attempted to determine whether WS-1 has the same effect *in vivo*. We experimentally infected newborn piglets with *E. coli* strain 4–1 after *B. subtilis* strain WS-1 treatment. As expected, the newborn piglets pre-inoculated with TSB medium containing *B. subtilis* strain WS-1 *via* oral feeding showed a lower diarrhea rate (16.7%) at the first day. By contrast, all newborn piglets (100%) pre-inoculated with TSB medium without *B. subtilis* strain WS-1 *via* oral feeding showed acute and severe watery diarrhea in the first 2 days (Figure 6A), indicating that *B. subtilis* strain WS-1 might serve as a probiotic in newborn piglets. Importantly, two piglets died that pre-inoculated with TSB medium without *B. subtilis* strain WS-1 *via* oral feeding group at 2 d.p.c., and no piglets died in the *B. subtilis* strain WS-1 treatment group during the study (Figure 6B). Taken together, these results demonstrated that *B. subtilis* strain WS-1 works as a probiotic to inhibit *E. coli* infection *in vivo*.

### Histopathological Results of Newborn Piglets Infected With *E. coli* After *B. subtilis* Strain WS-1 Treatment

**Figure 7:**
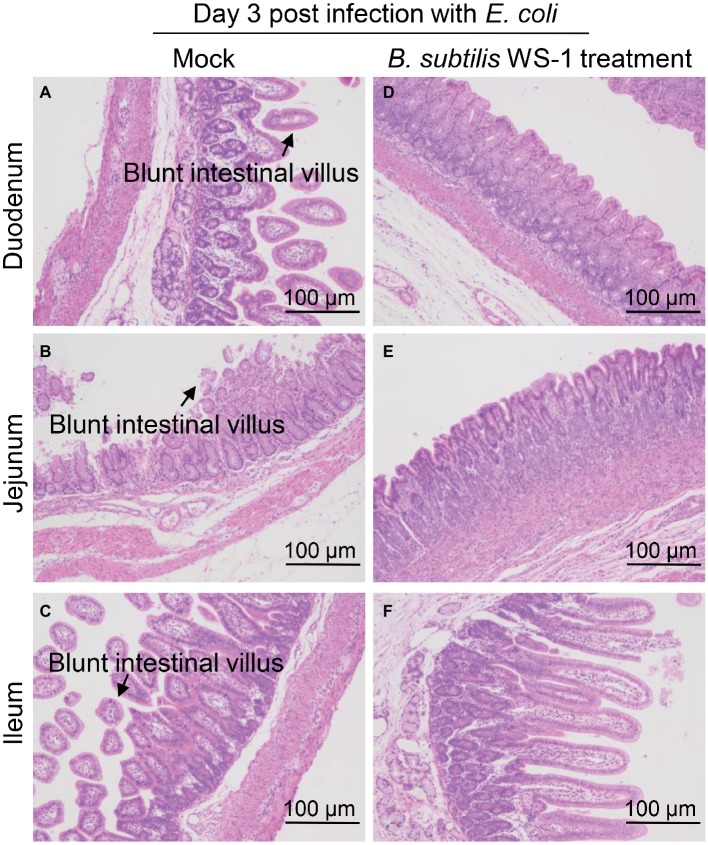
Intestinal changes in newborn piglets post-challenge with *E. coli* of the control group and the *B. subtilis* strain WS-1 treatment group. **(A–C)** Hematoxylin and eosin (H&E)-stained intestinal tissue section (blunt intestinal villus was indicated by arrows) of a control piglet at 3 d.p.c. **(D–F)** H&E-stained intestinal tissue section of a *B. subtilis* strain WS-1 treatment piglet at 3 d.p.c. The data are representative of two independent experiments. Data are represented as mean ± SD, *n* = 1 or *n* = 2.

To determine the histological changes in the intestine of newborn piglets infected with *E. coli* after *B. subtilis* strain WS-1 treatment, piglets were necropsied at 3 d.p.c. As shown in Figure 7, blunt intestinal villus was observed in the duodenum, jejunum, and ileum in the TSB medium only group, while the villus in the *B. subtilis* strain WS-1 treatment group remained intact (Figures 7A–F).

## Discussion

Since the first report of *B. subtilis* by Christian Gottfried Ehrenberg in 1835 (Sella et al., 2015), this ancient bacteria had been widely detected and isolated in many organisms and environments (Borsodi et al., 2011; Gu et al., 2015; Wei et al., 2016). Many subsequent studies demonstrated that *B. subtilis* had a variety of functions. Although *B. subtilis* was widely used in the field of fermentation and livestock and poultry breeding (Nguyen et al., 2015; Liu et al., 2017; Amorim et al., 2019), currently limited information is available regarding the protection of *B. subtilis* against intestinal diseases in newborn piglets. In the present study, we reported an isolate of *B. subtilis* from apparently healthy pigs in a pathogenic *E. coli* endemic commercial farm with severe diarrhea symptoms in piglets, which could inhibit the growth of *E. coli in vitro* and protect piglets from diarrhea and death caused by pathogenic *E. coli* in newborn piglets.

Diarrhea in piglets is mainly caused by viruses and bacteria such as Porcine epidemic diarrhea virus (PEDV) and *E. coli* (Vlasova et al., 2014; Garcia-Menino et al., 2018), which results in significant economic losses to the pig industry. Occasionally, some apparently healthy pigs without symptoms of diarrhea were found commingling with *E. coli* diarrheic piglets. To determine the protective agent in these pigs, 35 unknown bacterial strains from fresh feces of one healthy pig were isolated and 10 unknown bacterial strains were determined to markedly inhibit the growth of *E. coli* and *Salmonella typhi* (data not shown) by the Oxford cup method (Figure 1), indicating that these unknown strains might be the key to protect from diarrhea in these pigs. Of them, the No. 1 unknown bacterial strain had the best antibacterial effect. After analysis through conventional bacterial identification methods, the No. 1 unknown bacterial strain was identified as Gram-positive bacteria of *Bacillus*, having protease, lipase, and amylase activity, which might be utilized to generate antimicrobial substances such as bioactive peptides (Daroit et al., 2012) and chloramphenicol esters (Dong et al., 2017) to inhibit bacterial biofilm formation (Vaikundamoorthy et al., 2018). Bosshard et al. (2003) demonstrated that 95–99% similarity for 16S rRNA gene sequencing between two bacteria hints toward a similar species, while >99% indicates the same bacterium. In the study, the No. 1 unknown bacterial strain was identified as a member of *B. subtilis* by the analysis of 16S rRNA gene sequencing and therefore named as *B. subtilis* WS-1. To further characterize the isolate, the complete genome of *B. subtilis* strain WS-1 was sequenced and analyzed. The WS-1 strain shares 100% genomic synteny with *B. subtilis* and encoded multiple functional proteins like lipopeptides, which might be associated with the antibacterial activity of the WS-1 strain.

Bacterial resistance is becoming more common with the abuse of antibiotics over the past years, and it urgently demands an alternative to antimicrobial drugs. Of note, several groups have confirmed the protective role of *Bacillus* strains as probiotics *in vivo* (Wu et al., 2014; Ramesh et al., 2015). Collectively, these previous results confirmed that *Bacillus* strains could antagonize *Vibrio parahaemolyticus* and *Aeromonas hydrophila*. Swine enteric colibacillosis affects all sectors of the pig industry and all cycles of production. Compared with other age groups, newborn piglets are more vulnerable to swine enteric colibacillosis. We hypothesized that *B. subtilis* strain WS-1 was also capable of antagonizing colibacillosis in newborn piglets based on its probiotic effect *in vitro*. Therefore, we experimentally infected newborn piglets with the pathogenic *E. coli* after *B. subtilis* strain WS-1 treatment. As expected, *B. subtilis* strain WS-1 was able to work against diarrhea and death caused by *E. coli* in newborn piglets (Figure 6), indicating that the isolated *B. subtilis* strain WS-1 also had a probiotic effect *in vivo* and might be used as an alternative of antimicrobial drugs. In addition, we also found that none of the WS-1 strain-inoculated newborn piglets showed any clinical signs (data not shown) before *E. coli* infection, proving that *B. subtilis* strain WS-1 is safe for pigs. Furthermore, no evident gross lesions were observed in the intestinal tract of the *B. subtilis* strain WS-1 treatment piglets at necropsy at 3 d.p.c. (data not shown). Similarly, no microscopic lesions were observed in the small intestine in the *B. subtilis* strain WS-1 treatment group after *E. coli* infection (Figure 7), which furthered our understandings of the role of the *B. subtilis* as a probiotic. It was reported that several lipopeptides such surfactin, secreted by *B. subtilis*, can confer strong antipathogenic effects and thus benefit the host by balancing the intestinal microbiome (Zhou et al., 2018). Polyketide from the seaweed-associated heterotrophic bacterium *B. subtilis* MTCC 10403 has potential antibacterial activities against clinically important pathogens (Chakraborty et al., 2017). In addition, some studies concluded that some proteins involved in aggregation, such as flagella, might be associated with probiotic effects (Kleta et al., 2014). We determined that some genes in the WS-1 genome encode these proteins (Table 3). Whether the interference effect of WS-1 on *E. coli* infection in newborn piglets was associated with these putative proteins needs to be further explored. Nevertheless, there are still several important questions that need to be addressed. For example, what is the exact underlying mechanism of *B. subtilis* strain WS-1 inhibiting enteric diseases caused by *E. coli* in newborn piglets? Can *B. subtilis* strain WS-1 resist enteric diseases caused by viruses like PEDV in pigs? Does *B. subtilis* strain WS-1 have other applications apart from protecting the intestinal tract? Elucidation of these questions will elevate our understandings of the function of *B. subtilis* strain WS-1 and may help extend its application in many fields.

In summary, we isolated a field strain of *B. subtilis* from apparently healthy pigs growing with sick cohorts on one *E. coli* endemic commercial pig farm in Guangdong, China. Remarkably, inoculation of newborn piglets with 1.5 × 10^10^ CFU of WS-1 by oral feeding could prevent diarrhea and death caused by pathogenic *E. coli* in piglets. Collectively, these findings suggest that *B. subtilis* strain WS-1 has great potential to be explored as biocontrol agent protecting piglets from enteric diseases in pigs.

## Ethics Statement

The animal study was supervised by the Institutional Animal Care and Use Committee of Sun Yat-sen University (IACUC-2018-000178), and animals were used in accordance with regulations and guidelines of this committee.

## Author Contributions

YC and YD conceived and designed the experiments. YD, ZX, GY, WL, and DY performed the experiments. YD and ZX analyzed the data. YC, QZ, JL, LC, YZ, and CX contributed the reagents, materials, and analysis tools. ZX wrote the manuscript. YC checked and finalized the manuscript. All authors read and approved the final manuscript.

### Conflict of Interest Statement

The authors declare that the research was conducted in the absence of any commercial or financial relationships that could be construed as a potential conflict of interest.

## Footnotes

^1^http://www.megasoftware.net/

^2^http://www.megasoftware.net
